# Pediatric Cholesteatoma Presenting as Persistent Otorrhea: A Case of Delayed Diagnosis Across Multiple ENT Specialists

**DOI:** 10.1002/ccr3.73079

**Published:** 2026-06-29

**Authors:** And Elshani, Lorent Sijarina, Omar Alqaisi, Melisa Stublla, Liburn Grabovci, Melinda Hysenaj, Drilon Bytyçi, Anda Sylqa, Xhevdet Latifi, Shatha Al‐Sheyab, Patricia Tai

**Affiliations:** ^1^ Faculty of Medicine University of Prishtina Prishtina Kosovo; ^2^ Nursing Department Al‐Zaytoonah University Amman Jordan; ^3^ ENT Clinic University Clinical Center of Kosovo Prishtina Kosovo; ^4^ Radiology Department Mutah University Amman Jordan; ^5^ Department of Oncology University of Saskatchewan Saskatoon Saskatchewan Canada

**Keywords:** chronic otorrhea, conductive hearing loss, delayed diagnosis, mastoidectomy, pediatric cholesteatoma, temporal bone CT

## Abstract

Pediatric cholesteatoma is an aggressive but uncommon middle ear condition characterized by keratinizing squamous epithelium that can progressively erode adjacent structures. It is associated with faster bone destruction and higher recurrence rates in children than adults, and its symptoms often resemble chronic otitis media, which may delay diagnosis and treatment. A 4‐year‐old boy presented with persistent foul‐smelling otorrhea from the left ear and conductive hearing loss. Otoscopic examination revealed a retracted tympanic membrane with a whitish epitympanic mass. High‐resolution CT demonstrated soft‐tissue density in the middle ear with scutum erosion. The patient underwent canal wall‐up mastoidectomy with tympanoplasty, and intraoperative findings confirmed cholesteatoma with partial ossicular erosion. This case reflects the insidious clinical presentation of cholesteatoma and emphasizes that chronic otorrhea and unilateral hearing loss may indicate destructive middle ear disease rather than benign infection. Imaging plays a critical role in diagnosis and surgical planning, particularly in detecting bony erosion and disease extent. Maintaining a high index of suspicion for cholesteatoma in children with persistent discharge or unexplained conductive hearing loss is essential. Early diagnosis and timely surgical intervention are crucial to prevent irreversible complications and reduce recurrence risk.

## Key Clinical Message

Persistent unilateral, foul‐smelling otorrhea in children should prompt evaluation for cholesteatoma, especially when symptoms persist despite repeated ENT assessments. Early otoscopy plus targeted imaging (CT for surgical planning; DW‐MRI for follow‐up) enables timely surgery and prevents irreversible ossicular and intracranial complications.

## 1 Introduction

Cholesteatoma is a benign yet locally destructive epithelial lesion of the middle ear, characterized by keratinizing squamous epithelium that can progressively erode adjacent structures [1]. It typically arises either congenitally or as an acquired condition secondary to chronic middle ear disease. Globally, the annual incidence of pediatric cholesteatoma ranges from approximately 3 to 15 cases per 100,000 children, demonstrating a slight male predominance [2]. In acquired cases, chronic eustachian tube dysfunction and repeated infections lead to a retraction pocket in the tympanic membrane, allowing squamous epithelium to invade the middle ear space [2]. Over time, the cholesteatoma sac accumulates keratin debris and can enlarge, behaving like a self‐perpetuating cyst that produces enzymes causing bone destruction [3]. If undetected and left untreated, cholesteatoma may result in serious complications including ossicular chain erosion with conductive hearing loss, labyrinthine fistula, facial nerve palsy, and even intracranial extension [1, 2]. Early identification and surgical management are therefore critical to prevent irreversible morbidity from this “unsafe” ear disease [4, 5].

Clinically, cholesteatoma is often present insidiously. The most common initial presentation in the pediatric population consists of progressive conductive hearing loss and chronic, foul‐smelling otorrhea (ear discharge) that is typically refractory to standard medical therapies [2, 4]. Prompt diagnosis and intervention are paramount because the natural history of untreated cholesteatoma is progression to more extensive disease [3].

This case report exemplified the above principle. It describes a patient with chronic ear discharge in whom cholesteatoma eluded detection over multiple evaluations by ENT (ear, nose, throat) specialists, despite persistent otorrhea and hearing deficit.

## 2 Case Study

A 4‐year‐old boy presented with several weeks of persistent foul‐smelling discharge from the left ear. There was no history of prior otologic surgery, craniofacial anomalies, recent upper respiratory infection, fever, vertigo, or facial weakness. Growth and development were normal, and vaccinations were up to date.

On examination he was afebrile. Oropharyngoscopy and rhinoscopy were normal without signs of infection or adenoidal hypertrophy. Otoscopy of the left ear showed a retracted tympanic membrane with loss of light reflex and a whitish irregular mass arising from the superior external auditory canal and extending toward the tympanic cavity. The right ear had an intact, mobile tympanic membrane with no abnormalities.

Tympanometry demonstrated a type B curve on the left and type A on the right. Audiometry indicated conductive hearing loss in the affected ear. Ear swab culture showed no bacterial growth; microscopy revealed epithelial cells and scattered Gram‐positive cocci, likely contaminants.

Multiple prior ENT evaluations failed to identify the underlying pathology, likely due to the overlap with chronic otitis media with effusion and limited otoscopic visualization caused by recurrent inflammatory debris and poor cooperation in a distressed child. Limited access to advanced imaging and pediatric otologic equipment may have further contributed to the delay. Table 1 summarizes the timeline of care.

**TABLE 1:** Timeline of care.

Time point	Clinical presentation/event	Diagnostic & therapeutic interventions
Weeks 3–4 pre‐presentation	Initial onset of persistent, unilateral foul‐smelling left ear discharge	Symptomatic home care; parental observation
Weeks 1–2 pre‐presentation	Refractory otorrhea; progressive left‐sided conductive hearing loss	Multiple sequential outpatient ENT consultations; clinical otoscopy, audiometry, and ear swab culture
Day of presentation	Emergency referral to the otolaryngology clinic due to persistent symptoms	Comprehensive physical examination, tympanometry, and formal audiological assessment
Preoperative phase	Specialized diagnostic workup and surgical planning	High‐resolution temporal bone CT; routine laboratory evaluation (CBC, BMP, coagulation profiles)
Surgical intervention	Left middle ear and mastoid cholesteatoma excision under general anesthesia	Canal wall‐up (CWU) mastoidectomy with type III tympanoplasty; posterior canal wall reconstruction using cartilage and fascia grafts
Postoperative Days 1–2	Uncomplicated acute postoperative recovery	Intravenous antibiotic therapy (Ceftriaxone), local saline irrigation, and routine analgesia; discharged home on Day 2
Postoperative Day 7	Early surgical follow‐up	Retroauricular suture removal; verification of satisfactory wound healing
Week 3 follow‐up	Intermediate otologic evaluation	Otoscopic confirmation of graft integrity; resolution of otorrhea and subjective hearing improvement
Month 1 follow‐up	Short‐term treatment outcome assessment	Pure‐tone audiometry confirming partial improvement in conductive hearing thresholds
Months 6–12	Long‐term surveillance phase	Scheduled clinical otoscopy and non‐EPI diffusion‐weighted MRI (DW‐MRI) to monitor for recurrence

### 2.1 Laboratory and Imaging Evaluation

Preoperative evaluation included a complete blood count and a basic metabolic panel.

Laboratory tests showed a white blood cell count of 5.5 × 10^9^/L, hemoglobin of 11.3 g/dL, and mildly elevated C‐reactive protein of 7.6 mg/L, suggesting low‐grade inflammation. Coagulation profile, urea, creatinine, and glucose levels were within reference ranges.

High‐resolution CT of the temporal bone demonstrated fluid in the left tympanic cavity, scutum erosion, and irregular poorly defined ossicles. Mastoid air cells were pneumatized with partial effusion. These findings were consistent with chronic otomastoiditis and raised strong suspicion for cholesteatoma. The right temporal bone was normal with aerated mastoid cells and intact ossicles. No facial canal or inner ear dehiscence was identified (Figure 1).

**FIGURE 1:**
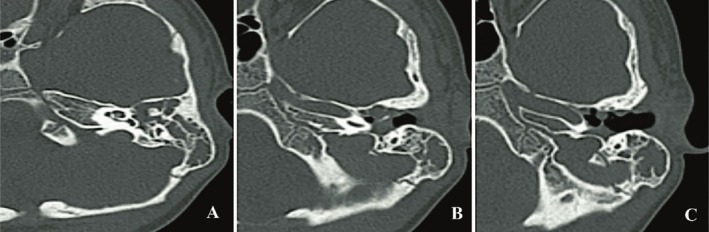
Selected axial high‐resolution computed tomography (HRCT) slices of the left temporal bone (A, B, and C). The images demonstrate a soft‐tissue density expanding Prussak's space, causing erosion of the scutum and ossicular chain, and extending into the completely obliterated left mastoid air cell network.

### 2.2 Surgical Management

Based on clinical and radiologic suspicion, the child underwent left mastoidectomy with type III tympanoplasty under general anesthesia. A retroauricular incision exposed the mastoid and middle ear cavity. Intraoperative findings revealed cholesteatoma tissue with polypoid extensions, which were completely excised (Figure 2).

**FIGURE 2:**
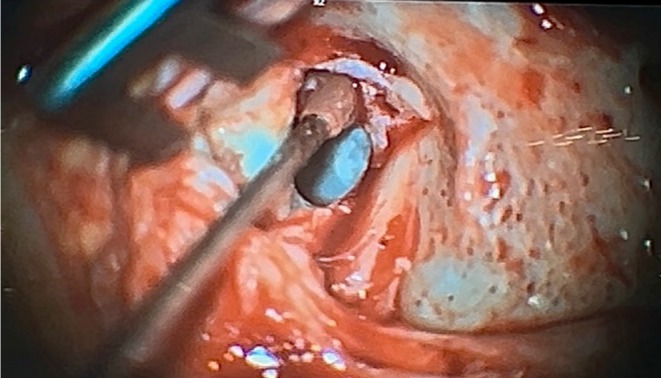
Intraoperative view of polypoid extensions arising from the attic of the tympanic membrane.

The ossicular chain was partially eroded with stapes discontinuity, although the footplate remained intact and mobile. Exploration of the mastoid antrum and aditus showed partial posterior canal wall erosion due to chronic inflammation and cholesteatoma invasion.

After complete disease removal, the posterior canal wall was reconstructed using temporalis fascia and cartilage grafts. The graft was secured, the tympanomeatal flap repositioned, and the canal packed with antibiotic‐soaked gauze. Hemostasis was achieved, and the wound closed in layers.

### 2.3 Postoperative Course

The postoperative course was uneventful. The patient received intravenous ceftriaxone for 7 days, saline ear irrigation, vitamin C, and diclofenac. Sutures were removed on Day 7; the patient had been discharged on Day 2 with home‐care instructions and 3 weeks' rest.

At three‐week follow‐up, the incision was healed, the canal dry and epithelialized, and the graft intact. There was no recurrent otorrhea, with reported improvement in hearing and speech. No facial nerve or vestibular deficits were present.

### 2.4 Outcome and Follow‐Up

At one‐month follow‐up, the patient was symptom‐free with complete resolution of discharge and intact graft healing. Audiologic testing showed partial improvement in conductive thresholds. Comparison of preoperative and postoperative pure‐tone audiometry demonstrated improvement in hearing thresholds of the affected ear (Figure 3). Follow‐up at 6 and 12 months is planned with clinical examination and diffusion‐weighted MRI to detect residual or recurrent cholesteatoma. Early surgery and complete disease removal likely prevented further ossicular damage and preserved hearing.

**FIGURE 3:**
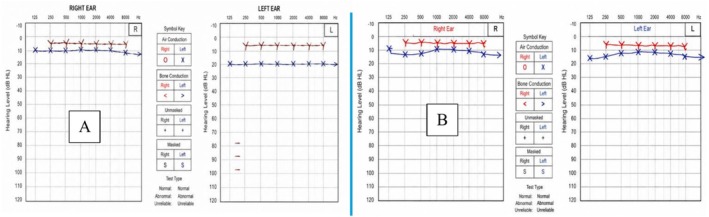
Comparison of preoperative (A) and postoperative (B) pure‐tone audiometry. Preoperative testing demonstrated conductive hearing loss in the left ear. Follow‐up audiometry performed 1 month after canal wall‐up mastoidectomy and type III tympanoplasty showed improvement in hearing thresholds, consistent with successful restoration of middle‐ear sound conduction.

## 3 Discussion

This case illustrates the typical presentation and successful management of acquired pediatric cholesteatoma. While sharing the same basic histopathological definition, pediatric cholesteatoma differs drastically from adult cholesteatoma in its biological behavior, anatomy, and clinical progression [6]. Pediatric lesions display a significantly more aggressive clinical course, characterized by rapid hyperproliferation of the keratinizing squamous epithelium and elevated enzymatic activity (such as matrix metalloproteinases), which accelerates the destruction of adjacent bony structures [7]. Furthermore, children exhibit highly pneumatized mastoid temporal bones with a richer vascularized mucosa compared to the sclerotic, poorly pneumatized mastoids typical of chronic adult disease [8]. This extensive air cell network provides low‐resistance pathways that facilitate the rapid, insidious spread of pediatric disease into the mastoid antrum, yielding higher post‐surgical recurrence rates [6, 9].

Cholesteatomas of the temporal bone and middle ear can be divided into [10]: congenital cholesteatoma, acquired cholesteatoma (98%), external ear canal cholesteatoma, mural cholesteatoma, and petrous apex cholesteatoma.

Acquired cholesteatoma typically results from chronic eustachian tube dysfunction and tympanic membrane retraction [5, 11]. Diagnosis is primarily clinical, supported by otoscopy and high‐resolution CT (HRCT) of the temporal bone to assess bony erosion and anatomical extent [12]. Diffusion‐weighted MRI (DW‐MRI) is highly specific for distinguishing cholesteatoma from other soft tissue masses and is crucial for postoperative surveillance [13].

The principal imaging differentials for middle‐ear soft‐tissue lesions include cholesteatoma, granulation tissue, and effusion; their distinguishing radiologic and clinical features are summarized in Table 2.

**TABLE 2:** Radiologic characteristics for differentiating cholesteatoma, granulation tissue, and middle‐ear effusion [7, 8, 9].

Characteristics	Cholesteatoma	Granulation tissue	Middle ear effusion
Bone erosion (CT)	Present	Absent	Absent
Restricted diffusion (non EPI DWI)	Yes	No	No
Contrast enhancement	Minimal/no	Yes	No [variable T2 hyperintensity; sometimes fluid levels]
Location	Prussak's space, mastoid	Post‐operation cavity. Inflamed mucosa	Middle ear cavity
Clinical course	Progressive, destructive	Reactive, inflammatory	Transient, resolves

Abbreviation: Non EPI DWI, Non‐Echo‐Planar Diffusion‐Weighted Imaging.

In pediatric patients, MRI is preferred to limit radiation exposure, although CT is often used for surgical planning and is more accessible in resource‐limited settings such as Kosovo in our case [6, 8].

Complete surgical excision is the only definitive treatment. Surgical options include canal wall‐up (CWU) and canal wall‐down (CWD) mastoidectomy. While CWU preserves anatomy, it carries a higher recurrence rate (20%–27%) compared to CWD (3%–11%) [14, 15]. Pediatric cholesteatoma shows recurrence rates ranging from approximately 16% to nearly 30%, notably higher than in adults, largely due to more aggressive disease biology and immature mastoid pneumatization [16].

Ossicular erosion, especially of the incus (~80%), is common and influences hearing outcomes. Long‐term follow‐up is essential. In the present case, a canal wall‐up approach was chosen to preserve normal anatomy and optimize hearing outcomes, despite its recognized risk of residual or recurrent disease [16]. High‐resolution CT was valuable for surgical planning, whereas non‐echo‐planar diffusion‐weighted MRI remains the preferred modality for postoperative surveillance but may be less accessible in resource‐limited settings [13]. Given the risk of recurrence and the potential impact of hearing loss on language development, long‐term audiologic and speech monitoring is recommended in pediatric patients [15]. MRI is recommended 6–12 months after canal wall‐up surgery if second‐look surgery is deferred, followed by annual imaging for 2–3 years or if symptoms recur. Non‐EPI DW‐MRI has replaced routine second‐look surgery in many centers as a highly sensitive, noninvasive method for detecting residual disease [17, 18].

While pediatric cholesteatoma profiles are well‐documented, this case report adds distinct clinical and educational value to the current literature by critically analyzing the diagnostic pitfalls that can occur across sequential specialized otolaryngology evaluations. Unlike many published reports that focus on late‐stage intracranial complications or overt congenital masses, our case highlights an aggressive, “silent” acquired lesion in a very young child that masterfully mimicked routine chronic otitis media with effusion. By detailing this multi‐specialist diagnostic delay and emphasizing the precise roles of high‐resolution CT (HRCT) for baseline structural mapping and non‐Echo‐Planar Diffusion‐Weighted MRI (non‐EPI DW‐MRI) for radiation‐free surveillance, this paper provides a highly practical clinical roadmap. It alerts community and specialized clinicians to maintain a low threshold for advanced imaging in refractory unilateral otorrhea, thereby minimizing diagnostic blind spots and preventing irreversible ossicular chain destruction.

## 4 Conclusion

- Consider cholesteatoma in any child with persistent foul‐smelling otorrhea or unexplained conductive hearing loss.
- Pediatric cholesteatoma grows faster and recurs more often (25%) than in adults
- HRCT is essential for surgical planning; DW‐MRI is the gold standard for follow‐up.
- Medical therapy fails; surgery is required to prevent complications like facial nerve palsy or intracranial abscess.
- The incus is the most frequently eroded ossicle (78%), directly impacting hearing.
- Postoperative surveillance for at least 3–5 years is mandatory to catch recurrence early.

## Author Contributions

**And Elshani:** conceptualization, data curation, investigation, writing – original draft. **Lorent Sijarina:** conceptualization, data curation, investigation, writing – original draft, writing – review and editing. **Omar Alqaisi:** conceptualization, data curation, investigation, writing – original draft, writing – review and editing. **Melisa Stublla:** conceptualization, data curation, investigation. **Liburn Grabovci:** data curation, investigation, writing – original draft. **Melinda Hysenaj:** conceptualization, data curation, investigation. **Drilon Bytyçi:** conceptualization, data curation, investigation. **Anda Sylqa:** conceptualization, data curation, investigation. **Xhevdet Latifi:** conceptualization, data curation, supervision, writing – original draft. **Shatha Al‐Sheyab:** conceptualization, data curation, investigation. **Patricia Tai:** conceptualization, data curation, investigation, supervision, writing – review and editing.

## Funding

The authors have nothing to report.

## Ethics Statement

Ethical approval was not required for this case report according to institutional policy.

## Consent

Written informed consent was obtained from the patient for the publication of this study and for using any images needed.

## Conflicts of Interest

The authors declare no conflicts of interest.

## Data Availability

Data sharing is not applicable to this article as no datasets were generated or analyzed during the current study.
